# Effect of c‐Ski on atrial remodelling in a rapid atrial pacing canine model

**DOI:** 10.1111/jcmm.14876

**Published:** 2019-12-09

**Authors:** Juan Wang, Min Han, Su‐xia Han, Cuiju Zhi, Suli Gao, Yao Li

**Affiliations:** ^1^ Department of Cardiology The Fifth Affiliated Hospital to Xin Jiang Medical University Urumchi Xin Jiang China; ^2^ Xin Jiang Medical University Urumchi Xin Jiang China; ^3^ Department of Cardiovascular Medicine Shanghai Pudong New Area People's Hospital Affiliated to Shanghai Health University Shanghai China

**Keywords:** atrial fibrillation, atrial fibrosis, c‐Ski, extracellular matrix, p38 MAPK, TGF‐β1/Smad signalling

## Abstract

Atrial fibrosis is an important factor in the initiation and maintenance of atrial fibrillation (AF); therefore, understanding the pathogenesis of atrial fibrosis may reveal promising therapeutic targets for AF. In this study, we successfully established a rapid atrial pacing canine model and found that the inducibility and duration of AF were significantly reduced by the overexpression of c‐Ski, suggesting that this approach may have therapeutic effects. c‐Ski was found to be down‐regulated in the atrial tissues of the rapid atrial pacing canine model. We artificially up‐regulated c‐Ski expression with a c‐Ski–overexpressing adenovirus. Haematoxylin and eosin, Masson's trichrome and picrosirius red staining showed that c‐Ski overexpression alleviated atrial fibrosis. Furthermore, we found that the expression levels of collagen III and α‐SMA were higher in the groups of dogs subjected to right‐atrial pacing, and this increase was attenuated by c‐Ski overexpression. In addition, c‐Ski overexpression decreased the phosphorylation of smad2, smad3 and p38 MAPK (p38α and p38β) as well as the expression of TGF‐β1 in atrial tissues, as shown by a comparison of the right‐atrial pacing + c‐Ski‐overexpression group to the control group with right‐atrial pacing only. These results suggest that c‐Ski overexpression improves atrial remodelling in a rapid atrial pacing canine model by suppressing TGF‐β1–Smad signalling and p38 MAPK activation.

## 1 INTRODUCTION

Atrial fibrillation (AF) is a supraventricular tachyarrhythmia characterized by uncoordinated atrial activation with consequent deterioration of atrial mechanical function that is associated with cardiovascular morbidity and increased mortality.^1^, ^2^ The development of AF is predicted by electrophysiological and structural changes that promote, propagate and maintain AF.^3^ Among the structural changes that occur in AF, atrial fibrosis is a hallmark of the AF‐induced structural remodelling that causes intra‐ and interatrial inhomogeneity in conduction, thus creating a substrate for local re‐entry and contributing to the progressive nature of AF.^4^, ^5^ To develop safer and more effective treatments for AF, a better understanding of its pathogenesis is urgently needed.

Cardiac fibrosis is characterized by the excessive deposition of extracellular matrix (ECM) proteins synthesized in the cardiac interstitium, and it contributes to both systolic dysfunction and diastolic dysfunction in many pathological cardiac conditions.^6^ Transforming growth factor β1 (TGF‐β1) is a powerful cytokine affecting a multitude of cellular functions, including differentiation, proliferation and apoptosis. It is a profibrotic cytokine that has been demonstrated to induce selective atrial fibrosis, suggesting its critical role in the pathogenesis of AF.^7^, ^8^ TGF‐β1 exerts its profibrotic effects primarily through phospho‐(p‐) smad2 and phospho‐smad4, and c‐Ski inhibits TGF‐β1 signalling through its interaction with these Smad proteins.^9^ c‐Ski, a homolog of the v‐Ski oncoprotein, is highly conserved across various species and is widely expressed in different tissues, including cardiac tissue.^10^ Cunnington *et al* demonstrated the antifibrotic properties of c‐Ski and its role in the regulation of cardiac myofibroblast phenotype and contractility.^11^ Liu *et al* showed that c‐Ski promotes skin fibroblast proliferation but decreases type I collagen, both of which have implications for wound healing and scar formation.^12^ In addition, our previous studies confirmed the repressive effect of c‐Ski on TGF‐β1–induced human cardiac fibroblast proliferation and ECM protein synthesis.^13^ Although these studies provided promising evidence in support of the involvement of c‐Ski in cardiac fibrosis, mechanistic data on the roles of these small molecules in AF and the associated atrial remodelling in animal models are still lacking.

In this study, we evaluated the effect of c‐Ski on atrial remodelling in a rapid atrial pacing canine model. We demonstrated that c‐Ski is significantly down‐regulated in the atrial tissues of the rapid atrial pacing canine model. We also observed that overexpression of c‐Ski inhibits atrial collagen accumulation and reverses AF‐induced atrial remodelling through the TGF‐β1–Smad pathway, suggesting that c‐Ski could be a promising target for the treatment of cardiac fibrosis and may play an important part in the atrial remodelling associated with AF.

## 2 MATERIALS AND METHODS

### 2.1 Rapid atrial pacing canine model

**Figure 1:**
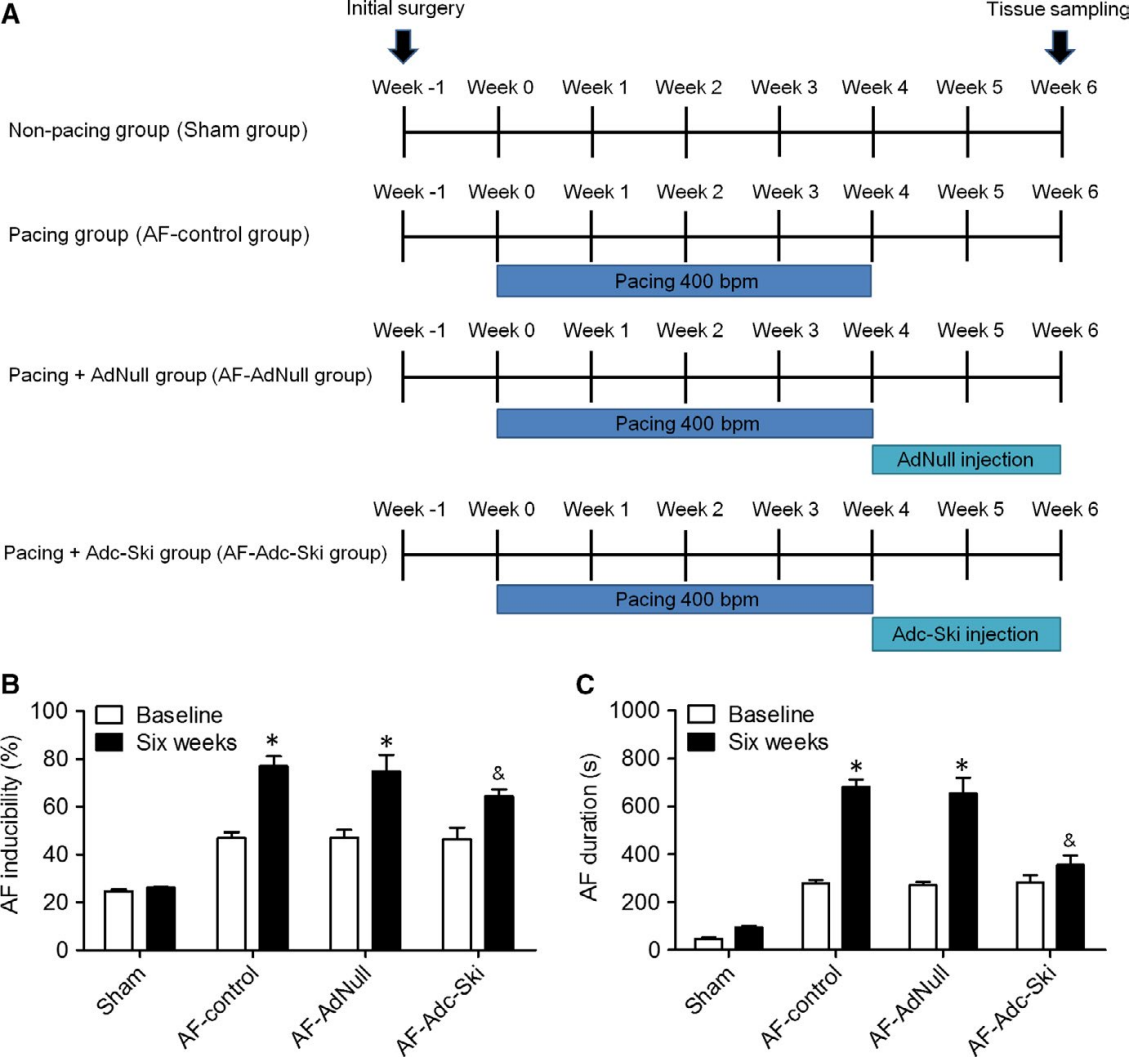
A schematic diagram of the study protocol and electrophysiological measurements. A, Observations started 1 week after the surgical procedure, and rapid atrial pacing was administered in the AF‐control, AF‐AdNull and AF‐Adc‐Ski groups for 4 weeks. Then, adenovirus was injected into multiple sites in the right atrium after atrial rapid pacing. B, The inducibility of AF was examined in the Sham, AF‐control, AF‐AdNull and AF‐Adc‐Ski groups. C, The duration of AF in the Sham, AF‐control, AF‐AdNull and AF‐Adc‐Ski groups. *P < .05 vs. the Sham group; ^&^ *P* < .05 vs. the AF‐control and AF‐AdNull groups

Adenovirus (Ad) expressing c‐Ski (Adc‐Ski) or a control transgene (AdNull) purchased from Hanbio (Shanghai, China) was used for the following in vivo experiments. Eighteen beagles weighing 10‐12 kg were obtained from Shanghai Jiagan Biotechnology Co., Ltd. (China) and were randomly divided into four groups: sham‐operated (Sham group, n = 3), atrial pacing control (AF‐control group, n = 3), atrial pacing and injected with AdNull (AF‐AdNull group, n = 6) and atrial pacing and injected with Adc‐Ski (AF‐Adc‐Ski group, n = 6). A programmable pacemaker was affixed to the backs of the dogs and attached to a pacing lead in the right atrium through the external jugular vein. The atrial pacing groups were subjected to continuous right‐atrial pacing at 400 bpm for 4 weeks before measurements (Figure 1A). The dogs in the Sham group were equipped in the same way but did not receive tachypacing. The dogs in the AF‐AdNull and AF‐Adc‐Ski groups were initially anaesthetized with 30 mg/kg sodium pentobarbital administered intravenously. A right‐side thoracotomy was performed in the first intercostal space. A pericardial cradle was created, and the adenovirus (200 μl of 5 × 10^9^ pfu/ml) was injected into multiple sites (~10 sites within a 1 cm^2^ area) of the right atrium. Then, a stimulus electrode consisting of five pairs of electrodes was hooked onto the injection site of the right atrium. On post‐gene transfer day 14, the animals underwent electrophysiological, histological and molecular analyses (Figure 1A). The animals were maintained in accordance with the guiding principles of the NIH Guide for the Care and Use of Laboratory Animals. The animal experiments were approved by the Experimental Animal Administration Committee of Shanghai Pudong New Area People's Hospital affiliated to Shanghai Health University.

### 2.2 Electrophysiological measurements

Electrophysiological measurements were obtained using an Electrophysiology Laboratory System with standard ablation catheters. The inducibility and duration of AF were measured at basic pacing cycle lengths of 300 ms AF induction was defined as P‐wave disappearance and rapid atrial activation with an irregular ventricular response on an atrial electrocardiogram after atrial programmed stimulation (S1 to S2), which was attempted three times at each site. The duration of induced AF was also recorded.^8^

### 2.3 Histopathology and immunohistochemistry

Atrial tissues collected from the dogs were fixed in a 4% paraformaldehyde solution, embedded in paraffin and sectioned into 5 μm slices. The resultant tissue slides were hydrated in a graded series of ethanol (100%, 95% and 75%) for 15 min each. Then, the slides were stained with haematoxylin and eosin (HE), Masson's trichrome and picric acid‐Sirius red to evaluate the presence of interstitial collagen fibre accumulation, indicative of cardiac fibrosis. Immunohistochemical assays for collagen III and α‐SMA were also performed using appropriate antibodies.

### 2.4 Quantitative reverse‐transcription polymerase chain reaction (qRT‐PCR)

Total RNA was isolated from atrial tissues performed with TRIzol reagent (Invitrogen, Carlsbad, CA, USA) according to the manufacturer's protocol. Complementary DNA synthesis was performed with the PrimeScript Reverse Transcriptase Reagent Kit (Takara, Dalian, China). qRT‐PCR was carried out on an Applied Biosystems 7900 Realtime PCR System with SYBR Green PCR Master Mix (Applied Biosystems, Foster City, CA, USA). *GAPDH* was used as an endogenous control for target normalization. Relative expression levels were determined by the 2^−ΔΔCT^ method. All assays were performed in triplicate.

### 2.5 Western blot analysis

Total protein was extracted from the atrial tissues of dogs for immunoblotting analysis. The protein content was determined by using the BCA Protein Assay Kit (Bio‐Rad, Hercules, CA, USA). Each protein sample was separated by 12% SDS‐PAGE and transferred to a polyvinylidene difluoride membrane (Millipore, Bedford, MA, USA). The membrane was incubated overnight at 4°C with the following primary antibodies: anti–c‐Ski (Abcam, Cambridge, MA, USA), anti–collagen III (Abcam), anti–α‐SMA (Abcam), anti–TGF‐β1 (Abcam), anti–total smad2/3 (Cell Signaling Technology [CST], Beverly, MA, USA), anti‐p‐smad2 (CST), anti–p‐Smad3 (CST), anti‐p38 (CST), anti‐p38α (CST), anti‐p38β (CST), anti‐p‐p38α (CST), anti‐p‐p38β (CST) and anti–p‐p38 (CST) antibodies. On the next day, the membrane was incubated with secondary antibodies diluted in PBS. Finally, the membrane was rinsed with PBS before scanning on an Infrared Imaging System (LI‐COR Biosciences). β‐Actin was used as an internal control for equal protein. Western blot bands were quantified by measuring the intensity of sample bands from each group and normalizing them to β‐actin intensity.

### 2.6 Statistical analysis

All data are presented as the mean ± SD. Statistical analyses were performed using GraphPad Prism 5.0 (GraphPad Software, USA). Two groups were compared using Student's *t* test, and more than two groups were compared by one‐way ANOVA. A *P* value less than 0.05 was considered statistically significant.

## 3 RESULTS

### 3.1 Electrophysiological measurements

The study protocol is summarized in Figure 1A. All dogs underwent echocardiography before the surgical procedure, and each dog was allowed to recover for 1 week after the procedure without atrial pacing to ensure stable baseline conditions. The study included 18 dogs subdivided into four groups: the Sham (n = 3), AF‐control (n = 3), AF‐AdNull (n = 6) and AF‐Adc‐Ski (n = 6) groups. The dogs from the AF‐control, AF‐AdNull and AF‐Adc‐Ski groups were subjected to continuous right‐atrial pacing at 400 bpm for 4 weeks after the aforementioned 1‐week recovery. A pericardial cradle was created, and the adenovirus was injected into multiple sites in the right atrium. The animals were killed 2 weeks after adenovirus injection. Electrophysiological measurements were obtained to determine the inducibility and duration of AF. As shown in Figure 1B and 1, the inducibility and duration of AF were much greater in the AF‐control and AF‐AdNull groups than in the Sham group and were lesser in the AF‐Adc‐Ski group than in the AF‐control and AF‐AdNull groups at 6 weeks after the start of the experiment.

### 3.2 c‐Ski expression in the atrial tissues of the rapid atrial pacing canine model

**Figure 2:**
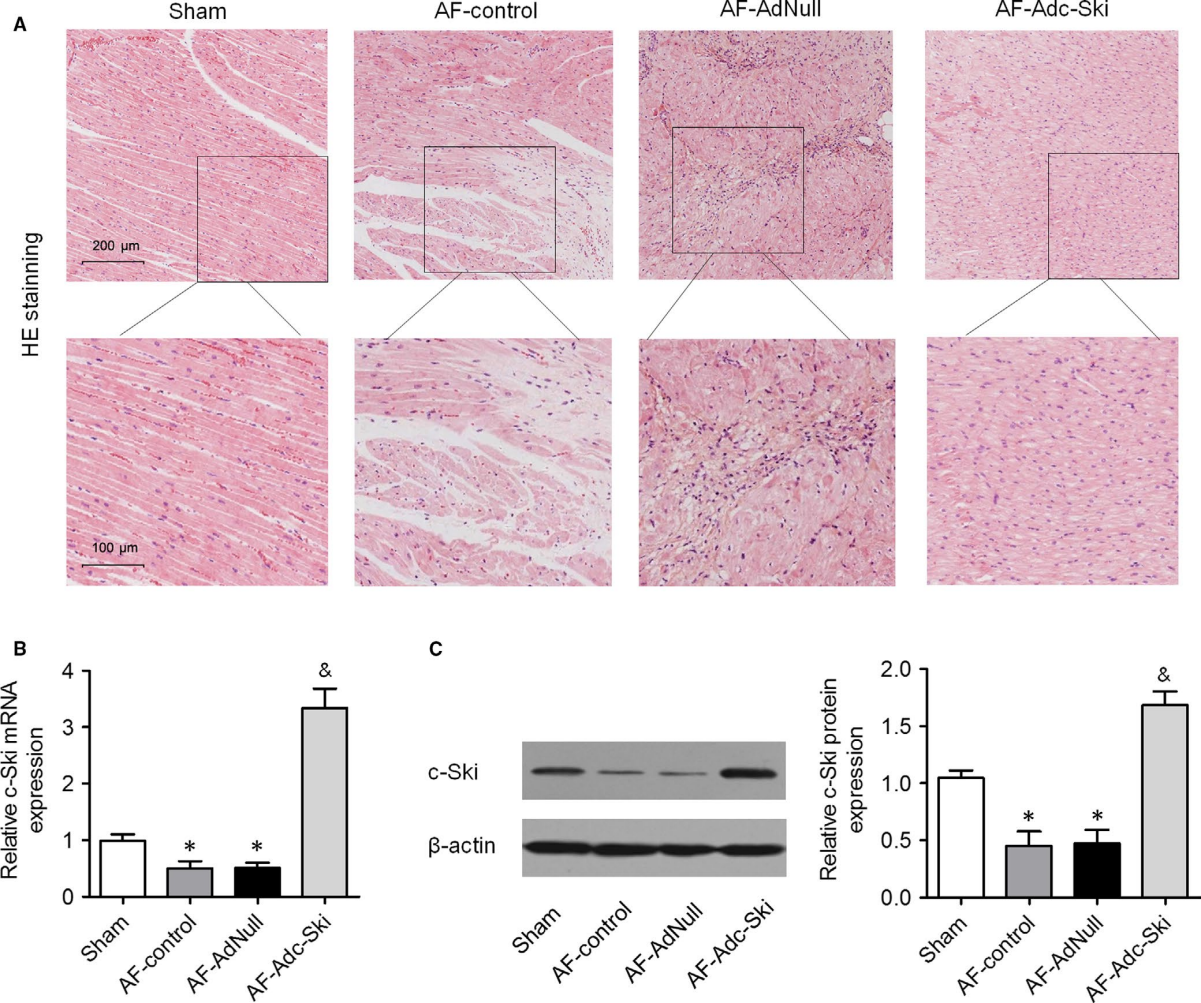
c‐Ski expression is low in the atrial tissues of rapid atrial pacing model. The dogs were randomly distributed into four groups: the sham‐operated (Sham, n = 3), atrial pacing control (AF‐control, n = 3), atrial pacing and injected with AdNull (AF‐AdNull, n = 6) and atrial pacing and injected with Adc‐Ski (AF‐Adc‐Ski, n = 6) groups. A, Representative images of the myocyte injuries in atrial tissues. B, c‐Ski mRNA expression in atrial tissues was measured in the four groups by qRT‐PCR. C, c‐Ski protein expression in atrial tissues was evaluated by Western blotting. *P < .05 vs. the Sham group; ^&^ *P* < .05 vs. the AF‐control and AF‐AdNull groups

Figure 2A presents the atrial histological changes observed by HE staining. This staining showed interstitial proliferation, misalignment and size irregularity in the muscle fibres of the AF‐control and AF‐AdNull groups when compared to the Sham group. These atrial tissue abnormalities were attenuated in the Ad‐Adc‐Ski group. To determine the role of c‐Ski in AF, c‐Ski levels in the atrial tissues from dogs in the four groups were examined by qRT‐PCR and Western blot assays. The results showed that c‐Ski mRNA and protein levels were significantly lower in the AF‐control and AF‐AdNull groups than in the Sham group but were obviously highest in the AF‐Adc‐Ski group (Figure 2B and 2). These results suggested that c‐Ski may play an important role in AF.

### 3.3 The effect of c‐Ski on fibrosis in the canine atrium

**Figure 3:**
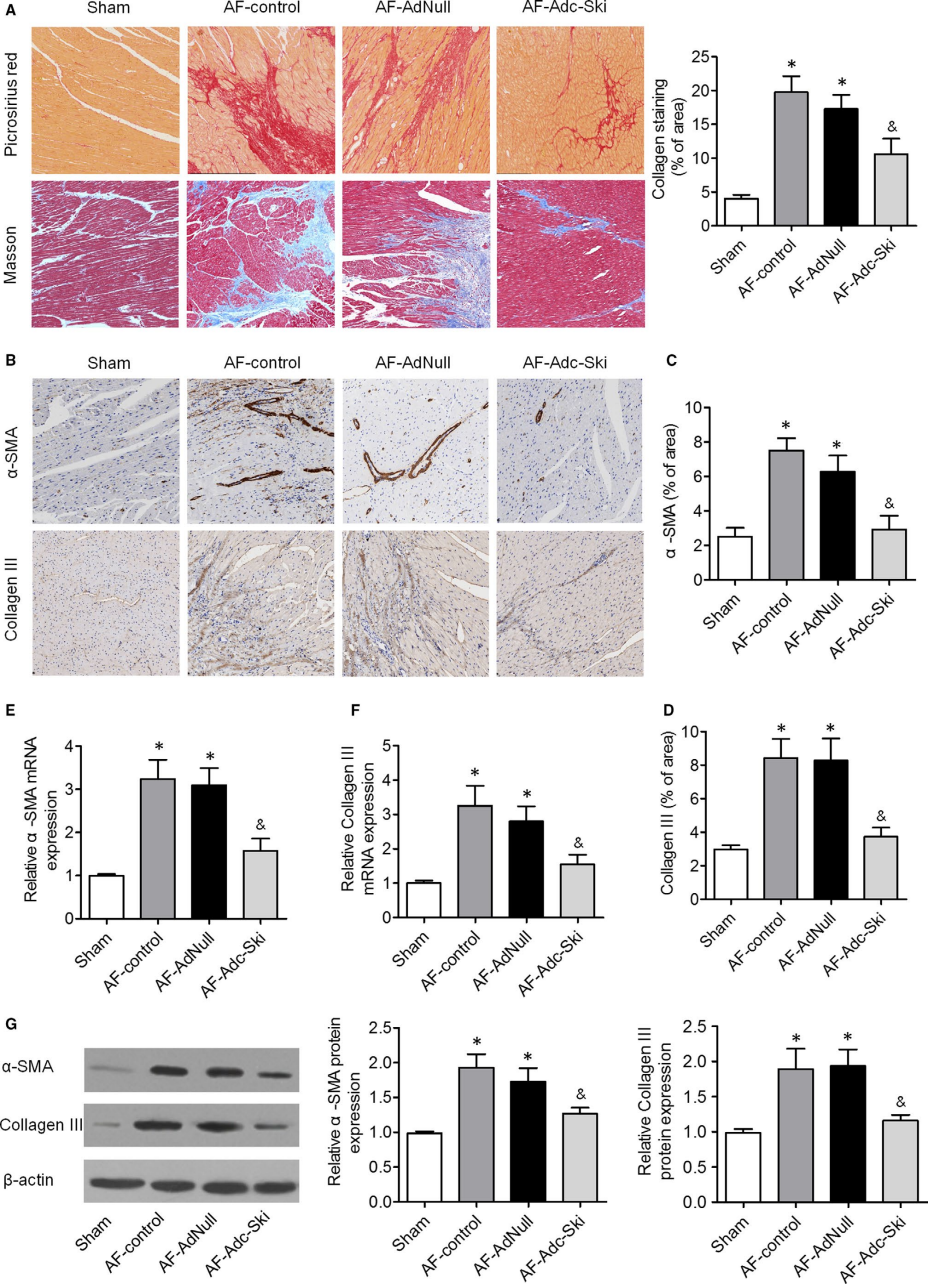
Masson's trichrome and picrosirius red staining and ECM expression in the atrial tissues of the four groups. A, Representative images of the atrial tissues of the myocardium following picrosirius red and Masson's trichrome staining in the Sham, AF‐control, AF‐AdNull and AF‐Adc‐Ski groups. B, Immunostaining for α‐SMA and collagen III in the four groups. C and D, Histograms presenting quantitative analyses of α‐SMA and collagen III staining. E and F, The mRNA expression levels of α‐SMA and collagen III in the atrial tissues of the four groups were measured by qRT‐PCR. G, The α‐SMA and collagen III protein levels in the atrial tissues of the four groups were evaluated by Western blotting. *P < .05 vs. the Sham group; ^&^ *P* < .05 vs. the AF‐control and AF‐AdNull groups

To assess the role of c‐Ski in structural remodelling, we systematically assessed atrial fibrosis in the four groups. Histopathological and immunohistochemical analyses were performed to determine the degree of fibrosis in the canine atrium. Masson's trichrome staining and picric acid–Sirius red staining revealed more extensive interstitial fibrosis in the AF‐control and AF‐AdNull groups than in the Sham group, and these abnormalities in the atrial tissues were attenuated in the AF‐Adc‐Ski group (Figure 3A). Immunohistochemical analysis showed that the expression levels of α‐SMA and collagen III were obviously higher in the atrial tissues of the AF‐control and AF‐AdNull groups than in the Sham group, and this increase was strongly attenuated in the atrial tissues of the AF‐Adc‐Ski group (Figure 3B‐D). The qRT‐PCR and Western blot results confirmed the significant increases in α‐SMA and collagen III expression in the AF‐control and AF‐AdNull groups when compared to the Sham group; this increase was attenuated by c‐Ski overexpression (Figure 3E‐G). In addition, we also showed that ED‐A fibronectin (ED‐A FN), platelet‐derived growth factor (PDGF)‐α and smooth muscle myosin heavy chain (SMemb) expression were obviously enhanced in AF‐control and AF‐AdNull groups; this change was attenuated by c‐Ski overexpression (Figure S1A‐C). These results indicated that c‐Ski overexpression improves atrial remodelling in a rapid atrial pacing canine model.

### 3.4 The influence of c‐Ski on TGF‐β1–Smad signalling and the p38 pathway in the rapid atrial pacing canine model

**Figure 4:**
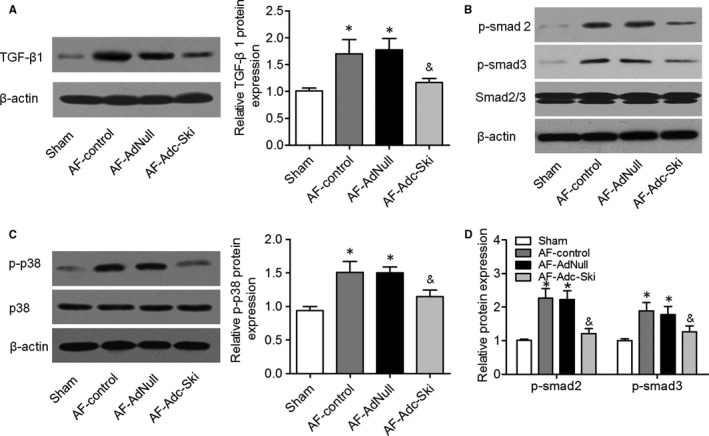
c‐Ski suppressed TGF‐β–SMAD signalling and the p38 pathway in the rapid atrial pacing canine model. A, TGF‐β1 protein expression levels in the atrial tissues of the Sham, AF‐control, AF‐AdNull and AF‐Adc‐Ski groups were assessed by Western blotting. B and C, The amounts of p‐smad2 and p‐smad3 and total smad2 and smad3 in the four groups were assessed by Western blotting. D, Western blots of p‐p38 and total p38. *P < .05 vs. the Sham group; ^&^ *P* < .05 vs. the AF‐control and AF‐AdNull group

Although the effect of the TGF‐β1–Smad pathway on the development of AF‐induced atrial fibrosis has been well documented,^14^ it is not clear whether c‐Ski regulates the TGF‐β–Smad axis in a rapid atrial pacing canine model. We measured TGF‐β1 protein expression in the atrial tissues of dogs from all four groups by Western blotting. The results showed that TGF‐β1 expression was higher in the atrial tissues of the AF‐control and AF‐AdNull groups than in the Sham group, and this up‐regulation was attenuated by c‐Ski overexpression (Figure 4A). We also demonstrated that the phosphorylation levels of smad2 (p‐smad2) and smad3 (p‐smad3) were significantly higher in the atrial tissues of the AF‐control and AF‐AdNull groups than in the Sham group, and c‐Ski overexpression reversed these increases in p‐smad2 and p‐smad3 (Figure 4B and 4). According to the Western blot results, the amount of p‐p38 was increased in the AF‐control and AF‐AdNull groups, and this increase was reduced in the AF‐Adc‐Ski group (Figure 4D). The p38 MAP kinase α and β isoforms (p38α and p38β) phosphorylation levels were examined in the atrial tissues of four groups, and the results showed that p‐p38α and p‐p38β levels were increased in the AF‐control and AF‐AdNull groups, and this increase was reduced in the AF‐Adc‐Ski group (Figure S2A‐C). Therefore, c‐Ski overexpression alleviated AF‐induced atrial fibrosis, possibly by inhibiting TGF‐β1–Smad signalling and the p38 MAPK pathway.

## 4 DISCUSSION

Atrial fibrillation is a clinical condition present in various cardiovascular diseases that has substantial effects on mortality and morbidity. Accordingly, AF is an important public health problem. In our study, we successfully established a rapid atrial pacing canine model and found that the inducibility and duration of AF were significantly reduced by the overexpression of c‐Ski, suggesting that this approach could have therapeutic effects. We also determined that c‐Ski was significantly down‐regulated in the atrial tissues of the canine model, and we successfully increased c‐Ski levels with a c‐Ski–expressing adenovirus. We demonstrated that c‐Ski overexpression alleviated atrial fibrosis by HE, Masson's trichrome and picrosirius red staining. We next showed that the expression levels of collagen III and α‐SMA were higher in the AF‐control and AF‐AdNull groups, and these increases were reversed by c‐Ski overexpression. In addition, comparison of the AF‐Adc‐Ski group to AF‐control and AF‐AdNull groups showed that c‐Ski attenuated the phosphorylation of smad2, smad3, and p38α and p38β and the expression of TGF‐β1 expression in atrial tissues. These findings revealed that c‐Ski overexpression improves atrial remodelling in this canine model, probably by suppressing both TGF‐β1–Smad signalling and p38 MAPK activation.

Extensive evidence confirms that structural remodelling, particularly interstitial fibrosis, is a major contributor to the physical basis of AF.^15^, ^16^ Thus, understanding the pathogenesis of cardiac fibrosis may identify promising targets for the treatment of patients with AF. Studies also suggest that the c‐Ski protein plays an important role in the development of nerve and muscle as well as in organ fibrosis.^17^, ^18^, ^19^ Recently, researchers have focused on the effects of c‐Ski in cardiac fibrosis. For example, Wang *et al* reported that miR‐155 regulates the TGF‐β–induced endothelial‐mesenchymal transition of human coronary artery endothelial cells via c‐Ski, thereby affecting cardiac fibrosis.^20^ Zhang *et al* found that c‐Ski can ameliorate isoproterenol‐induced myocardial fibrosis in a rat model and reduce both TGF‐β1–induced proliferation of primary rat cardiac fibroblasts and ECM deposition.^19^ Our team previously determined the involvement of c‐Ski in the regulation of TGF‐β–induced proliferation of human cardiac fibroblasts and ECM protein expression.^13^ Experimental studies in animal models revealed that atrial fibrosis plays an important role in the induction and maintenance of AF. We successfully established a rapid atrial pacing canine model and found that the inducibility and duration of AF were significantly reduced by the overexpression of c‐Ski, suggesting that this approach could have therapeutic value. We observed that c‐Ski expression was low in the atrial tissues of the model canines, and we increased c‐Ski expression with a c‐Ski–overexpressing adenoviral vector. Cardiac fibrogenesis is mainly characterized by the accumulation and deposition of ECM proteins, especially type I collagen, and increased levels of ECM proteins may be directly involved in the pathogenesis of AF.^21^ In our study, HE, Masson's trichrome and picrosirius red staining confirmed that c‐Ski overexpression alleviated atrial fibrosis. In addition, c‐Ski overexpression obviously reduced the increase in collagen III and α‐SMA levels induced by rapid atrial pacing. Li *et al* showed that the pathophysiological changes in different regions of the atria were not uniform in right and left atrial fibrosis. Specifically, no difference in fibrosis was observed between the six different parts of the right atrium, whereas a significant difference in fibrosis was observed between six different parts of the left atrium.^22^ They also observed that fibrosis was more severe in the left atrium than in the right atrium.^22^ Major limitations remain in our knowledge of the pathological and electrophysiological mechanisms underlying AF, which require further study.

The TGF‐β signalling pathway is one of the central regulatory systems in cardiac fibrogenesis,^23^, ^24^ and c‐Ski has been identified as an inhibitor of TGF‐β signalling, which functions through its interaction with Smad proteins.^9^, ^25^ Here, we observed that c‐Ski overexpression decreased the phosphorylation of smad2 and smad3 and the expression of TGF‐β1 in atrial tissues, based on a comparison of the levels in the AF‐Adc‐Ski group to those in the AF‐control and AF‐AdNull groups. Several studies have shown that p38 MAPK is activated in various forms of heart failure and induces fibrotic remodelling in the myocardium.^26^ Two well‐characterized isoforms of p38 MAPK (p38α and p38β) share extensive sequence similarity and a broad range of tissue distribution, such as the heart tissue, and function as important roles in cardiac hypertrophy.^27^, ^28^, ^29^ Furthermore, collagen can directly increase pulmonary‐vein cardiomyocyte arrhythmogenesis through the activation of p38 MAPK, which may contribute to the pathogenesis of AF.^21^ We also demonstrated that c‐Ski overexpression decreased AF‐induced p38 MAPK phosphorylation (including p‐p38α and p‐p38β). These findings suggest that c‐Ski overexpression improves atrial remodelling in a canine model with rapid atrial pacing, probably by suppressing both TGF‐β1–Smad signalling and p38 MAPK activation.

The major finding of this study is that overexpression of c‐Ski in vivo alleviates atrial fibrosis. To the best of our knowledge, this study is the first to show the therapeutic effects of c‐Ski overexpression in a rapid atrial pacing canine model. We demonstrated that c‐Ski was underexpressed in the atrial tissues of our canine model. In addition, we observed that overexpression of c‐Ski suppressed the accumulation of atrial collagen and reversed AF‐induced atrial remodelling probably by inhibiting TGF‐β1–Smad signal transduction and the p38 MAPK pathway. These data suggest that c‐Ski may be a promising target for the treatment of cardiac fibrosis and may perform important functions in the atrial remodelling associated with AF.

## CONFLICT OF INTEREST

The authors confirm that there are no conflicts of interest.

## AUTHORS CONTRIBUTIONS

Juan Wang, Min Han and Su‐xia Han designed the experiment and wrote the manuscript; Cuiju Zhi and Juan Wang analysed the data; Min Han, Suli Gao and Yao Li did the experiments. All authors read and approved the final manuscript.

## Data Availability

Research data are not shared. The data that support the findings of this study are available on request from the corresponding author. The data are not publicly available due to privacy or ethical restrictions.
