# Supplementary material for: Effect of c‐Ski on atrial remodelling in a rapid atrial pacing canine model
Source: J Cell Mol Med. 2019 Dec 9;24(2):1795–803. doi: 10.1111/jcmm.14876 (PMC6991632; doi:10.1111/jcmm.14876)
Supplement: Supplementary file 1 [file JCMM-24-1795-s001.docx]

**SUPPLEMENTARY FIGURE 1**


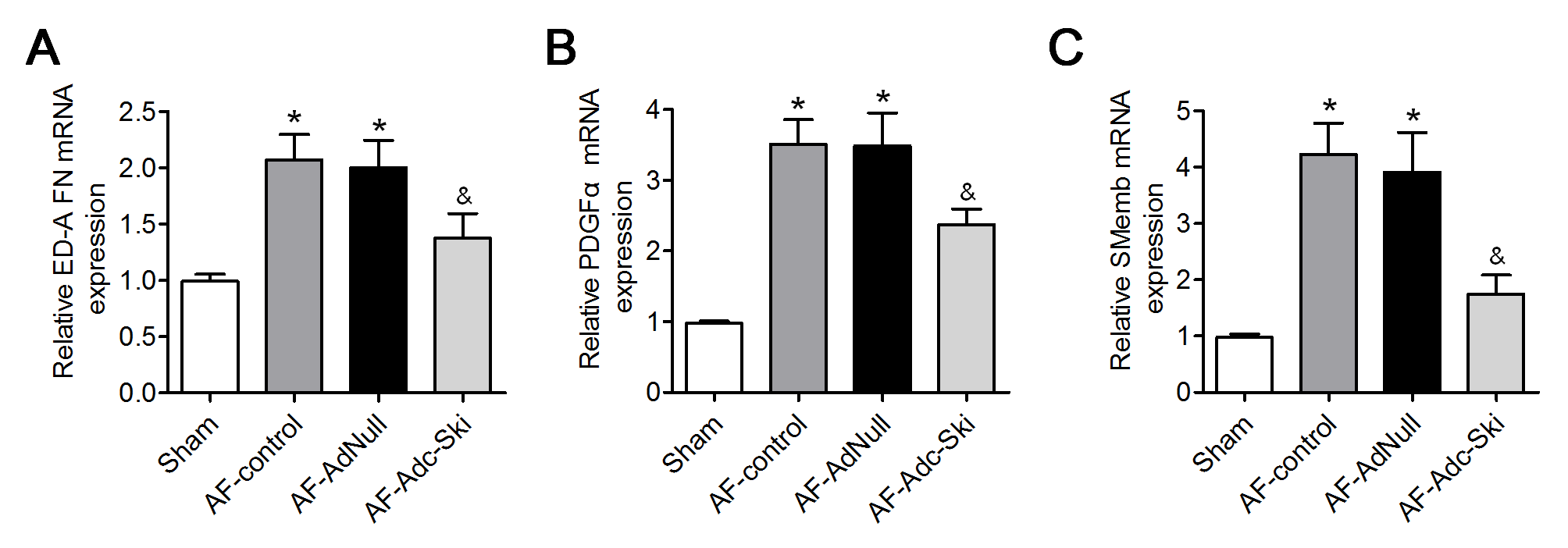


SUPPLEMENTARY FIGURE 1. The mRNA expression levels of ED-A FN (A), FDGFα (B) and Smemb (C) in the atrial tissues of the four groups were measured by qRT-PCR. ^*^*P* < 0.05 vs. the Sham group; ^&^*P* < 0.05 vs. the AF-control and AF-AdNull groups.

**SUPPLEMENTARY FIGURE 2**


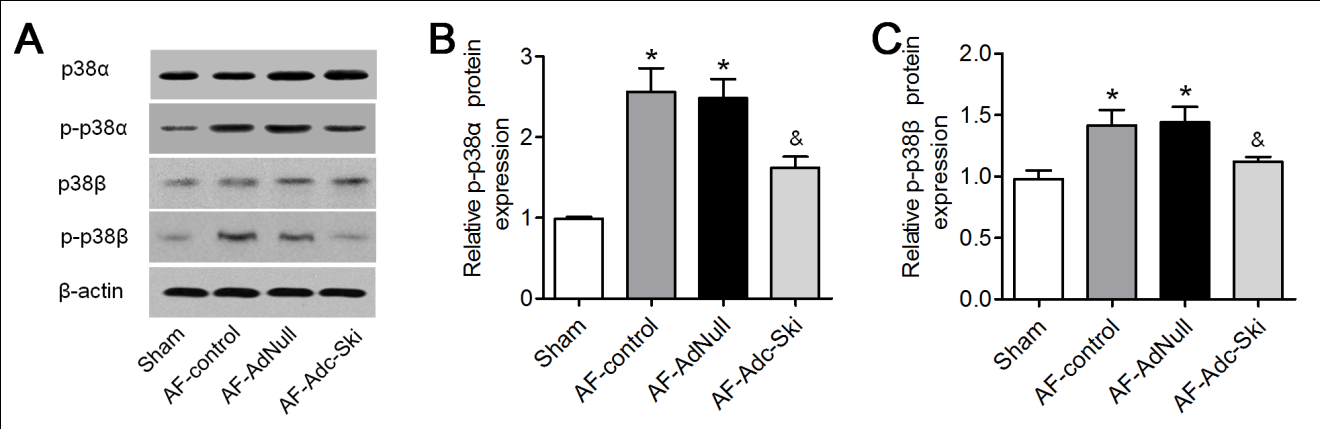


SUPPLEMENTARY FIGURE 2. (A-C) The p38 MAP kinase α and β isoforms (p38α and p38β) phosphorylation levels in the atrial tissues of the four groups were measured by qRT-PCR. ^*^*P* < 0.05 vs. the Sham group; ^&^*P* < 0.05 vs. the AF-control and AF-AdNull groups.
